# Xanomeline–Trospium Validates Muscarinic Agonism as an Effective Non-Dopaminergic Treatment for Schizophrenia

**DOI:** 10.3390/ijms27135734

**Published:** 2026-06-25

**Authors:** Ghaith K. Mansour, Ahmad W. Hajjar, Adnan H. Hajjar, Abdullah Alissa, Hatouf H. Sukkarieh

**Affiliations:** 1 Department of Pharmacology, College of Medicine, Alfaisal University, Riyadh 11533, Saudi Arabia; gkmansour@alfaisal.edu (G.K.M.); aaalissa@alfaisal.edu (A.A.); 2 College of Medicine, Alfaisal University, Riyadh 11533, Saudi Arabia; awhajjar@alfaisal.edu; 3 Department of Internal Medicine, East Carolina University Medical Center, Greenville, NC 27834, USA; hajjara24@ecu.edu

**Keywords:** schizophrenia, muscarinic receptors, xanomeline, trospium chloride, antipsychotic therapy

## Abstract

Schizophrenia remains a debilitating global health challenge where pharmacologic treatment has been stagnant for over seventy years, relying almost exclusively on the blockade of dopamine receptors. While this mechanism controls positive psychosis, it frequently fails to address negative symptoms or cognitive impairment and carries a significant burden of metabolic and motor adverse effects. This review evaluates the scientific and clinical validation of xanomeline–trospium (Cobenfy^®^; investigational name KarXT), the first approved antipsychotic with a completely non-dopaminergic mechanism of action. We synthesize data ranging from the unique structural biology of the bitopic M1/M4 muscarinic receptor agonist xanomeline to the pharmacokinetic innovation of using the peripheral antagonist trospium chloride to mitigate systemic toxicity. Comprehensive analyses of the EMERGENT clinical trial program demonstrate that this combination significantly reduces heterogeneous schizophrenia symptoms with a safety profile distinct from current standards of care, specifically avoiding weight gain and extrapyramidal movement disorders. Furthermore, we contrast this success with the recent failures of other novel mechanisms and explore the potential for precision medicine through the identification of muscarinic receptor deficit biotypes. We conclude that M1/M4 muscarinic receptor agonism represents an important advance toward circuit-based therapeutics that may help overcome some of the limitations inherent to dopamine-centered pharmacotherapy.

## 1. Introduction: The Stagnation and Resurgence of Psychiatric Drug Development

Schizophrenia is a chronic, severe mental disorder that affects approximately 24 million people globally, translating to a prevalence of roughly 0.32% or 1 in 300 individuals [1,2,3,4]. The disorder typically onsets in late adolescence or early adulthood [5,6], disrupting critical developmental milestones in education, social functioning, and vocational attainment. Consequently, schizophrenia is a leading cause of years lived with disability worldwide [7]. The impact extends far beyond the clinical symptoms. Individuals with schizophrenia exhibit a reduction in life expectancy of 10 to 25 years [8,9,10]. This excess mortality is driven partly by suicide, particularly in the early stages of illness, but predominantly by comorbid non-communicable diseases such as cardiovascular disease, metabolic syndrome, and diabetes [7].

Economically, the burden is immense. In the United States, the annual societal cost of schizophrenia was estimated at nearly $343 billion in recent analyses [11,12]. A striking feature of this economic landscape is that direct healthcare costs, such as hospitalizations, medications, and outpatient care, account for only a fraction of the total. The majority of the burden stems from indirect costs, including unemployment, reduced productivity, premature mortality, and the immense uncompensated labor provided by family caregivers [11]. This distribution underscores a critical failure of existing treatments: while they may manage acute crises, they frequently fail to restore the functional capacity necessary for patients to return to the workforce and reintegrate into society [8].

Since the serendipitous discovery of chlorpromazine in the 1950s, the “dopamine hypothesis” has served as the foundational bedrock of antipsychotic drug development [13,14,15]. This theory posits that the symptoms of schizophrenia arise from a dysregulation of dopaminergic transmission: specifically, a hyperdopaminergic state in the mesolimbic pathway driving positive symptoms (hallucinations, delusions) and a hypodopaminergic state in the prefrontal cortex contributing to negative symptoms (anhedonia, avolition) and cognitive deficits [13]. Guided by this hypothesis, every antipsychotic agent approved prior to late 2024 shared a common primary mechanism of action: antagonism or partial agonism of the dopamine 2 receptor (D2) [16,17,18,19], although several agents, most notably clozapine, also exert clinically significant actions at non-dopaminergic targets, including muscarinic, serotonergic, and histaminergic receptors [8,20]. While this approach has provided relief from psychosis for millions, it has reached a ceiling of efficacy. Approximately 30% of patients meet the criteria for treatment-resistant schizophrenia, failing to respond to at least two adequate trials of antipsychotics [21,22].

For these patients, clozapine remains the only evidence-based option, yet its use is limited by the risk of agranulocytosis and severe metabolic burden [8,20,23]. Furthermore, D2 blockade does not effectively treat, and may even worsen, the negative and cognitive symptom domains [24]. These symptoms are the strongest predictors of long-term functional outcome and quality of life, yet they remain largely refractory to current pharmacotherapy [8]. The safety profile of dopaminergic agents also presents significant challenges, ranging from the extrapyramidal symptoms [25,26,27] and tardive dyskinesia associated with high-potency first-generation antipsychotics (typical antipsychotics) to the weight gain, dyslipidemia, and insulin resistance characteristic of many second-generation agents (atypical antipsychotics) [8].

Modern neuroscience has moved beyond the reductionist view of schizophrenia as a single-neurotransmitter deficit [28,29,30]. It is now conceptualized as a disorder of dysregulated neural circuitry involving complex interactions between dopamine, glutamate, gamma-aminobutyric acid, and acetylcholine (ACh) [19]. The “glutamate hypothesis”, centered on N-methyl-D-aspartate receptor (NMDAR) hypofunction, offers a more comprehensive explanation for the cognitive and negative symptoms, implicating a disruption in the excitation/inhibition balance within the prefrontal cortex [19].

Within this network-based framework, the cholinergic system has emerged as a master regulator. Postmortem and neuroimaging studies have consistently identified reductions in muscarinic acetylcholine receptor density, particularly the M1 and M4 subtypes, in the hippocampus, prefrontal cortex, and striatum of individuals with schizophrenia [31]. These receptors are strategically positioned to modulate the very circuits disrupted in the disorder. M4 receptors in the midbrain and striatum can inhibit dopamine release presynaptically, offering a non-dopaminergic “brake” on psychosis [24]. Concurrently, M1 receptors in the cortex can drive the activity of parvalbumin (PV)-positive interneurons, potentially restoring cognition [32]. Thus, targeting muscarinic receptors represents a shift from symptom suppression to circuit modulation. This review critically examines the approval of xanomeline–trospium (Cobenfy^®^; formerly known by the investigational name KarXT during clinical development by Karuna Therapeutics, subsequently acquired by Bristol-Myers Squibb) as a landmark validation of a non-dopaminergic antipsychotic approach. By synthesizing insights from structural biology, the EMERGENT clinical program, and evolving precision medicine frameworks, we evaluate muscarinic modulation not merely as a novel mechanism, but as a significant advance toward circuit-based therapeutics that addresses key limitations of dopamine-centered pharmacotherapy.

## 2. Neurobiological Basis of Schizophrenia: A Circuit-Level Framework

Schizophrenia is no longer conceptualized as a disorder of isolated neurotransmitter excess or deficiency but as a disorder of dysregulated neural circuitry. The cortico-striato-thalamo-cortical (CSTC) loop “the principal circuit governing sensorimotor gating, reward processing, and cognitive function” is disrupted at multiple nodes in schizophrenia [27,28]. Understanding this circuit-level failure is essential for appreciating why muscarinic M1/M4 receptor agonism, which targets multiple nodes within this circuit simultaneously, represents a mechanistically coherent therapeutic strategy.

### 2.1. The CSTC Circuit and Dopaminergic Imbalance

**Figure 1:**
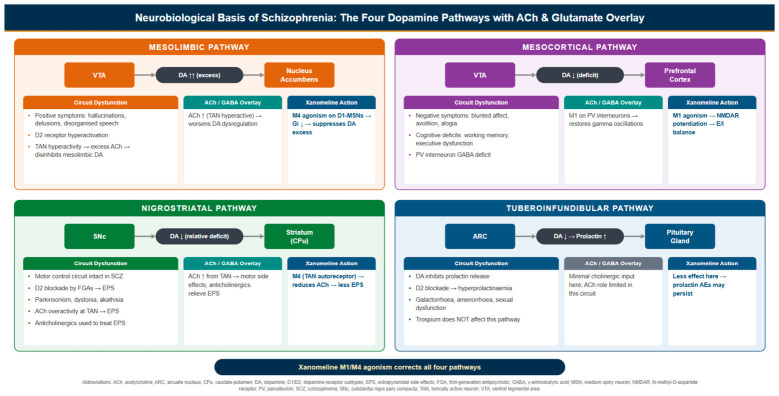
Neurobiological basis of schizophrenia: The four dopamine pathways with acetylcholine and glutamate overlay. Schizophrenia involves circuit-specific dysregulation across four major dopaminergic projections. The mesolimbic pathway (VTA → nucleus accumbens) exhibits dopamine excess, driving positive symptoms via D2 receptor hyperactivation; concomitant TAN hyperactivity floods the striatum with acetylcholine, which overwhelms inhibitory M4 signaling and amplifies dopamine overflow. The mesocortical pathway (VTA → dlPFC) shows dopamine deficiency, impairing working memory and executive function, compounded by PV interneuron GABA deficit that disrupts cortical gamma oscillations. The nigrostriatal pathway (SNc → striatum) is relatively preserved in schizophrenia but is vulnerable to iatrogenic dopamine depletion by D2-blocking antipsychotics, producing extrapyramidal side effects worsened by TAN-mediated acetylcholine excess. The tuberoinfundibular pathway (arcuate nucleus → pituitary) is disrupted by antipsychotic D2 blockade, removing tonic inhibition of prolactin secretion and causing hyperprolactinemia. Xanomeline, a preferential M1/M4 muscarinic agonist, addresses multiple circuit abnormalities simultaneously: M4 agonism on striatal D1-MSNs and TAN autoreceptors suppresses mesolimbic dopamine excess and reduces acetylcholine-driven EPS risk; M1 agonism on cortical PV interneurons and pyramidal neurons potentiates NMDAR currents, restores gamma oscillatory activity, and improves cognitive function. Trospium, the peripherally confined quaternary ammonium co-administered in the fixed-dose combination (Cobenfy^®^), does not cross the blood–brain barrier and exerts no central effect.

The CSTC loop encompasses projections from the ventral tegmental area (VTA) and substantia nigra pars compacta to the striatum (dorsal and ventral), which project via the globus pallidus and subthalamic nucleus to the thalamus, which relays back to the prefrontal cortex (PFC) and other cortical regions. Dopaminergic dysregulation in this circuit represents the best-characterized pathophysiological signature of schizophrenia [13,28]. In the mesolimbic subdivision (VTA to nucleus accumbens), dopamine is hyperactive, disrupting sensorimotor gating and driving positive symptoms [13]. Conversely, in the mesocortical subdivision (VTA to PFC), dopamine is relatively deficient, impairing working memory, executive function, and goal-directed behavior, generating the negative and cognitive symptom domains [8,24]. Critically, striatal cholinergic interneurons (tonically active neurons, TANs) serve as a fundamental modulatory gate within the CSTC circuit. M4 muscarinic autoreceptors on TANs inhibit acetylcholine release, modulating the influence of cholinergic signaling on dopaminergic terminals. This positions the M4 receptor as an endogenous homeostatic brake within the CSTC circuit, deficient in schizophrenia due to reduced M4 receptor expression [31], which xanomeline directly restores (Figure 1).

### 2.2. Parvalbumin Interneurons and the Gamma Oscillation Deficit

A second major node of CSTC circuit dysfunction involves parvalbumin (PV)-positive GABAergic interneurons in the dorsolateral prefrontal cortex (dlPFC) [33,34]. PV interneurons provide perisomatic inhibition to pyramidal neurons, orchestrating the high-frequency (30–80 Hz) gamma oscillations essential for working memory, attention, and information integration [35]. In schizophrenia, PV interneuron function is profoundly disrupted: expression of the GABA-synthesizing enzyme GAD67 is reduced, parvalbumin protein levels are diminished, and the protective perineuronal nets (ECM scaffolds stabilizing fast-spiking PV function) are degraded [33,34]. These molecular deficits impair PV interneuron firing rates and temporal precision, collapsing gamma oscillatory synchrony and producing the cognitive impairments characteristic of schizophrenia. M1 muscarinic receptors are densely expressed on PV interneurons in the dlPFC [31,32]. Activation of M1 receptors depolarizes PV cells, increasing their excitability and firing frequency; a mechanism by which xanomeline’ s M1 agonism may restore gamma oscillations and cognitive function [32].

### 2.3. The NMDAR/Glutamate Hypothesis and Its Cholinergic Intersection

The glutamate/NMDA receptor (NMDAR) hypothesis provides a complementary framework of particular relevance to understanding xanomeline’s mechanism. The hypothesis originates from the observation that NMDAR antagonists such as phencyclidine (PCP) and ketamine reproduce the full spectrum of schizophrenia symptoms in healthy subjects, including positive, negative, and cognitive features, more completely than dopaminergic agents alone [15]. The primary pathophysiological mechanism involves NMDAR hypofunction on fast-spiking PV interneurons, which are particularly sensitive to NMDAR antagonism due to their high expression of GluN2A and GluN2D subunits. This selectively impairs GABAergic inhibitory tone, producing a paradoxical disinhibition of glutamatergic pyramidal neurons—a chaotic, desynchronized cortical excitatory state that drives the excitation/inhibition (E/I) imbalance central to cognitive impairment in schizophrenia [15].

Critically, the cholinergic and glutamatergic systems intersect rather than run parallel. M1 muscarinic receptor activation potentiates NMDAR-mediated synaptic currents in pyramidal neurons through a protein kinase C (PKC)-dependent phosphorylation mechanism that enhances NMDAR trafficking to the synapse [19]. This means that xanomeline’s M1 agonism does not merely stimulate PV interneurons directly but also functionally opposes NMDAR hypofunction in pyramidal neurons, providing a molecular explanation for its pro-cognitive effects that complements the PV interneuron restoration model described above. Agents targeting NMDAR function via the glycine co-agonist site (e.g., the GlyT1 inhibitor iclepertin) demonstrated cognitive benefits in Phase 2 but failed to improve the MATRICS Consensus Cognitive Battery composite score in three independent Phase 3 CONNEX trials [36]. This trajectory contrasts with xanomeline–trospium, which showed consistent separation from the placebo across three Phase 3 studies, and may reflect the advantage of targeting the cholinergic gateway to NMDAR function; a more physiologically nuanced route to restoring E/I balance. Together, CSTC circuit disruption, PV interneuron dysfunction, and NMDAR hypofunction converges on a unified framework: schizophrenia is a disorder of cortical network dysregulation in which cholinergic M1 and M4 receptor deficits serve as critical links between upstream dopaminergic imbalance and downstream cognitive-behavioral impairment. Restoring muscarinic M1/M4 signaling therefore targets multiple nodes of this network simultaneously.

## 3. Medicinal Chemistry and Formulation Science

Xanomeline is the core active pharmaceutical ingredient driving the efficacy of the new class. Chemically, it is identified as 3-(3-hexyloxy-1,2,5-thiadiazol-4-yl)-1,2,5,6-tetrahydro-1-methylpyridine [37,38]. Physiochemically, xanomeline tartrate presents as a white to slightly tan crystalline solid. Its solubility profile is notable for being highly soluble in protic solvents such as water and methanol, as well as polar organic solvents like dimethylformamide and dimethyl sulfoxide. However, it exhibits poor solubility in lipophilic solvents like octanol and hexane [39]. Trospium chloride is a quaternary ammonium antimuscarinic agent [40,41,42]. It appears as white crystalline in the form of powder [43]. Like xanomeline, it is highly soluble in water and methanol but practically insoluble in methylene chloride [44]. The defining structural feature of trospium is its permanent positive charge (quaternary ammonium), which renders it highly hydrophilic. It cannot cross the blood–brain barrier [45]. Unlike tertiary amine antimuscarinics (e.g., oxybutynin), which can cause central nervous system effects, trospium remains restricted to the peripheral compartment [46]. This pharmacokinetic compartmentalization is the linchpin of the xanomeline–trospium (Cobenfy^®^) strategy: trospium occupies peripheral muscarinic receptors, blocking the adverse effects of xanomeline without interfering with its central therapeutic action [47].

## 4. Pharmacodynamics and Neurobiology

### 4.1. Muscarinic Receptor Subtypes and Signaling

Muscarinic receptors are GPCRs that mediate the metabotropic actions of acetylcholine [48,49]. They are classified into five subtypes (M1–M5) [50], which differ in their G-protein action type [51]. M1, M3, and M5 (Gq/11-coupled): These receptors couple to Gq proteins, activating phospholipase C to generate diacylglycerol and inositol triphosphate. This cascade triggers intracellular calcium mobilization and protein kinase C activation, generally resulting in increased neuronal excitability [51].

**Figure 2:**
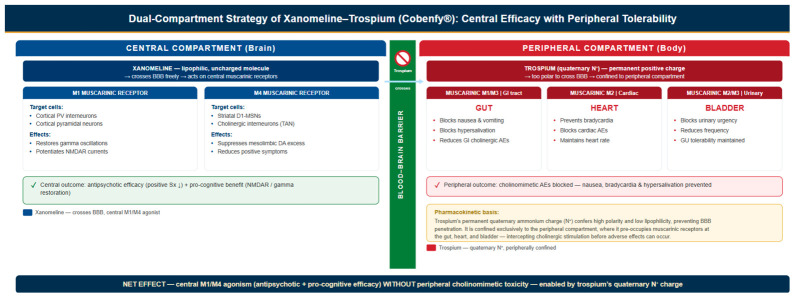
Dual-compartment strategy of xanomeline–trospium (Cobenfy^®^): Central efficacy with peripheral tolerability. Xanomeline–trospium exploits compartment-specific pharmacokinetics to separate therapeutic from adverse effects. Xanomeline (lipophilic, uncharged) crosses the BBB freely, engaging central M1 receptors on cortical PV interneurons and pyramidal neurons—restoring gamma oscillations and potentiating NMDAR currents—and M4 receptors on striatal D1-MSNs and TANs, suppressing excess mesolimbic dopamine release. Trospium (quaternary ammonium, permanent N^+^ charge) cannot cross the BBB and remains confined to the periphery, pre-occupying muscarinic receptors in the gut (M1/M3; blocking nausea and hypersalivation), heart (M2; preventing bradycardia), and bladder (M2/M3; reducing urgency), thereby intercepting the cholinomimetic adverse effects that previously precluded xanomeline monotherapy. The net result is central M1/M4 agonism—antipsychotic and pro-cognitive efficacy—without peripheral cholinomimetic toxicity. Abbreviations: BBB, blood–brain barrier; D1-MSN, direct-pathway medium spiny neuron; M1–M4, muscarinic receptor subtypes; NMDAR, N-methyl-D-aspartate receptor; N^+^, quaternary ammonium charge; PV, parvalbumin; TAN, tonically active neuron.

M2, M4 (Gi/o-coupled): These receptors couple to Gi/o proteins, inhibiting adenylyl cyclase and reducing the cyclic adenosine monophosphate (cAMP) levels [51,52]. Muscarinic receptors also activate G-protein-gated inwardly rectifying potassium channels, leading to membrane hyperpolarization [49]. Xanomeline binds to all five muscarinic receptor subtypes, with the highest activity at the M1 and M4 receptor subtypes (Figure 2) [38,53].

### 4.2. Bitopic Binding Mode, Receptor Affinity, and Wash-Resistant Engagement

**Figure 3:**
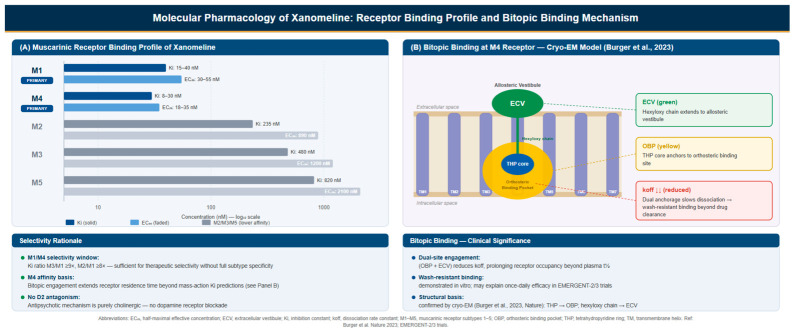
Molecular pharmacology of xanomeline: Muscarinic receptor binding profile and bitopic binding mechanism at the M4 receptor. (**A**) Xanomeline demonstrates preferential affinity at M1 (Ki 15–40 nM; EC_50_ 30–55 nM) and M4 (Ki 8–30 nM; EC_50_ 18–35 nM) muscarinic receptor subtypes—designated primary therapeutic targets—with 8–50-fold lower affinity at M2, M3, and M5 subtypes, conferring a selectivity window that permits central target engagement without meaningful peripheral muscarinic activation; notably, xanomeline exerts no dopamine D2 receptor antagonism. (**B**) Based on cryo-EM structural data [33], xanomeline engages the M4 receptor through a bitopic mechanism in which the tetrahydropyridine (THP) core occupies the orthosteric binding pocket (OBP, yellow), while the hexyloxy side chain simultaneously extends to the extracellular vestibule (ECV, green), an allosteric site structurally divergent across subtypes; this dual-site anchorage reduces the dissociation rate constant (k_off ↓↓), producing wash-resistant receptor occupancy that persists beyond plasma drug clearance and underlies the enhanced M4 subtype selectivity.

Standard orthosteric agonists bind only to the acetylcholine site. In contrast, xanomeline engages the M4 receptor through a bitopic binding mode, interacting with both the orthosteric pocket and an adjacent extracellular allosteric site [33,53]. This dual interaction slows dissociation and produces wash-resistant receptor engagement, which may help explain why its effects outlast its plasma half-life of about 5 h [34]. This prolonged receptor engagement may contribute to xanomeline’s efficacy duration relative to its plasma half-life of approximately 5 h (Figure 3).

Cognitive symptoms in schizophrenia are linked to reduced activity in the dorsolateral prefrontal cortex and impaired gamma oscillations, which are high-frequency neural rhythms important for attention and working memory [35]. These oscillations depend on synchronized parvalbumin-positive interneurons, which provide inhibitory control over pyramidal neurons [35]. In schizophrenia, PV interneurons are dysfunctional [54], leading to a breakdown of neural synchrony [35].

M1 receptors are densely expressed on these PV interneurons. Activation of M1 receptors depolarizes PV cells, increasing their excitability [32]. Additionally, M1 activation enhances N-methyl-D-aspartate receptor (NMDAR) currents in pyramidal neurons [19]. The antipsychotic efficacy of xanomeline is primarily attributed to M4 receptor activation. This subtype has been associated with the modulation of dopamine [24]. M4 receptors function as a physiological “brake” on this system via two mechanisms: (1) M4 receptors are located on striatal projection neurons (specifically D1-expressing MSNs), where their inhibitory Gi signaling counters the excitatory Gs signaling of D1 receptors [55]. (2) Acetylcholine stimulates dopamine release via nicotinic receptors on dopaminergic terminals. M4 receptors serve as auto receptors on these ACh-neurons; when activated, they inhibit acetylcholine release [56]. Importantly, this modulation preserves basal dopaminergic tone [24].

## 5. Pharmacokinetics and Pharmacogenomics

Following oral administration, xanomeline reaches peak plasma concentrations in approximately 2 h [57], while trospium peaks in about 4.5 h [41]. A crucial clinical consideration is the food effect. Administration with a high-fat meal reduces trospium Cmax by approximately 70% and AUC by 85% [39]. Consequently, xanomeline–trospium (Cobenfy^®^) must be administered at least one hour before or two hours after meals [39]. This dosing requirement may pose adherence challenges in the schizophrenia population, where medication compliance is already a recognized barrier to effective treatment, and should be addressed through patient education and clinical monitoring strategies. Xanomeline has an apparent volume of distribution of approximately 10,800 L, derived from population pharmacokinetic analyses reported in the FDA Clinical Pharmacology Review (NDA 216158) [39]. This large Vd (~154 L/kg) is consistent with xanomeline’s high lipophilicity and extensive CNS tissue binding. Trospium has a substantially smaller Vd of approximately 395–531 L, well-documented in the published pharmacokinetic literature [41], consistent with its confinement to the peripheral compartment due to its permanent quaternary ammonium charge [38]. Xanomeline undergoes extensive hepatic metabolism by CYP2D6, 2B6, 1A2, 2C9, and 2C19 and flavin-containing monooxygenases 1 and 3 [39].

Xanomeline also inhibits CYP3A4 and P-glycoprotein in the gastrointestinal tract, which may produce clinically relevant drug–drug interactions when co-administered with CYP3A4-sensitive substrates or P-glycoprotein substrates [39]. Given the high prevalence of polypharmacy in patients with schizophrenia, clinicians should carefully evaluate concomitant medications before initiating therapy. It has a mean elimination half-life of approximately 5 h [39]. In contrast, trospium is primarily excreted unchanged in the urine via active tubular secretion (85% of dose), with a half-life of roughly 6 h [39].

Genetic variations in CYP2D6 significantly impact xanomeline exposure. Population PK analyses indicate that intermediate metabolizers exhibit increased xanomeline exposure, while ultrarapid metabolizers show an approximate 43% reduction in Cmax and AUC compared to normal metabolizers. This reduction could theoretically compromise efficacy in ultrarapid metabolizers, although specific dose adjustments for this group are not currently mandated in the label. The pharmacokinetics in poor metabolizers have not been fully characterized, suggesting a need for clinical vigilance in this subgroup [58]. Due to the metabolic and excretory pathways involved, xanomeline–trospium is contraindicated in patients with moderate to severe hepatic impairment (Child–Pugh B and C) and moderate to severe renal impairment (eGFR < 60 mL/min) [58]. Given the high prevalence of metabolic comorbidities in patients with schizophrenia, including hepatic steatosis and diabetes-related nephropathy [8], these contraindications may limit the eligible patient population and warrant consideration in clinical practice. Hepatic or renal impairment leads to marked increases in xanomeline exposure, potentially increasing the risk of systemic toxicity [58].

## 6. Preclinical Safety and Toxicology

Long-term carcinogenicity studies have provided a robust safety dataset. In 2-year dietary studies in rats, xanomeline showed no increase in tumor incidence at doses up to 37 mg/kg/day (males) and 46 mg/kg/day (females), representing 1.4–1.8 times the maximum recommended human dose [39]. Trospium chloride also demonstrated no carcinogenic potential in rats at doses up to 32 times the maximum recommended human dose [39]. Both compounds tested negative in standard genotoxicity batteries, including the Ames test and in vivo micronucleus assays [39]. While not carcinogenic, high doses of xanomeline were associated with hepatobiliary changes in rats, including biliary hyperplasia, biliary cysts, and ductule dilatation [39]. These findings correlate with transient elevations in liver enzymes observed in some clinical trial participants, necessitating hepatic monitoring during treatment [35]. Safety pharmacology studies also assessed cardiovascular risk. While xanomeline inhibits the Human Ether-a-go-go-related Gene (hERG) channel in vitro (IC50 = 70 nM), a value that typically warrants careful evaluation, in vivo studies showed no QTc prolongation in thorough QT studies conducted as part of the regulatory submission, although dose-dependent increases in heart rate were observed [39,59].

## 7. Clinical Efficacy

The clinical validation of xanomeline–trospium (XT) was established through the EMERGENT program [60]. Three pivotal 5-week, randomized, double-blind, placebo-controlled trials (EMERGENT-1, -2, and -3) formed the basis of approval. These trials enrolled adults with acute psychosis, having PANSS (Positive and Negative Syndrome Scale) total scores of 80–120. It should be noted that all pivotal trials were placebo-controlled and conducted in inpatient settings; no direct head-to-head comparisons with active antipsychotic comparators have been published to date, which limits the ability to draw definitive comparative efficacy conclusions. Primary endpoint (PANSS Total): XT demonstrated a statistically significant reduction in PANSS total scores compared to the placebo. In EMERGENT-2, the least squares mean (LSM) difference was −9.6 points (*p* < 0.001); in EMERGENT-3, it was −8.4 points (*p* < 0.001) [58]. The effect sizes (Cohen’s d) ranged from 0.60 to 0.65; for context, a meta-analysis of standard-of-care antipsychotics reported pooled effect sizes in the range of 0.38–0.50 for acute schizophrenia trials [61]. Positive symptoms: XT significantly reduced the PANSS Positive Subscale scores (LSM difference −3.2; *p* < 0.0001) [61]. Negative symptoms: XT showed significant improvements in the PANSS Negative Subscale and Marder Negative Factor scores (LSM difference −1.7; *p* < 0.0001) [61]. Agitation: Post-hoc analyses using the PANSS-Excited Component (PANSS-EC) revealed significant reductions in agitation across all three trials (Cohen’s d = 0.43 to 0.62) [62].

The 52-week open-label extension study (EMERGENT-4) provided critical long-term efficacy data. Patients rolling over from the placebo arms of the acute trials experienced rapid improvements upon starting XT, while those maintained on XT showed continued symptom reduction. At the data cutoff, over 69% of participants who completed the 52-week trial achieved a ≥30% reduction in PANSS total scores, with a mean reduction of 33.3 points from the baseline [58,63]. These results should be interpreted cautiously, given the open-label design and the potential for enrichment bias among completers (Table 1).

Cognitive outcomes: Type 1 post hoc analyses from EMERGENT-1 using the Cogstate Brief Battery showed numerically greater improvement with xanomeline–trospium (XT) than the placebo (*p* = 0.16), although this was not statistically significant in the full sample. When the analysis was restricted to patients with clinically meaningful baseline cognitive impairment and after the exclusion of those with excessive performance variability, statistically significant cognitive benefits were observed [61]. These findings should be interpreted as hypothesis-generating rather than confirmatory, because the analyses were post hoc and cognitive endpoints were not prospectively powered [38]. EMERGENT-5 (second long-term open-label extension): The EMERGENT-5 study provides critical data on the durability of treatment response and long-term maintenance parameters. Patients continuing XT maintained robust symptom control with sustained Positive and Negative Syndrome Scale (PANSS) total score reductions [64]. New initiators (entering from placebo arms) demonstrated rapid onset of improvement consistent with the pivotal trials. Notably, GI adverse events attenuated with continued treatment—rates of nausea and vomiting were lower than in the acute 5-week trials—an important finding for long-term adherence. Heart rate elevation was stable and non-progressive. No new safety signals were identified over the extended observation period. EMERGENT-5 collectively establishes xanomeline–trospium as a viable long-term maintenance therapy, distinguishing it from drugs whose tolerability profiles lead to high attrition in real-world settings.

**Table 1:** The EMERGENT Clinical Trial Program.

EMERGENT-1	Phase 2 RCT, DB-PC	5 weeks	PANSS total score	LSM Δ −17.4 vs. −5.9 (*p* < 0.001). Proof-of-concept [33]
EMERGENT-2	Phase 3 RCT, DB-PC	5 weeks	PANSS total score	LSM Δ −9.6 (*p* < 0.001); Cohen’s d 0.60–0.65. FDA approval basis [59]
EMERGENT-3	Phase 3 RCT, DB-PC	5 weeks	PANSS total score	LSM Δ −8.4 (*p* < 0.001); Cohen’s d 0.60–0.65. FDA approval basis [59]
EMERGENT-4	Open-label extension	52 weeks	Long-term safety and efficacy	69% achieved ≥30% PANSS reduction; mean PANSS Δ −33.3 from baseline [59,65]
EMERGENT-5	Open-label extension	Long-term	Long-term safety and maintenance efficacy	Sustained PANSS improvement on maintenance therapy. GI adverse events attenuated over time (improved long-term tolerability). Heart rate elevation stable, not progressive. No new safety signals. Confirms viability as long-term maintenance option [PMID: 41868713].

DB-PC, double-blind placebo-controlled; LSM, least-squares mean; PANSS, Positive and Negative Syndrome Scale; RCT, randomized controlled trial.

## 8. Safety, Tolerability, and the Metabolic Advantage

The safety profile of xanomeline–trospium (XT) is distinct from all other antipsychotics. The most common treatment-emergent adverse events were constipation (21%), nausea (19%), dyspepsia (19%), and vomiting (14%) [47]. These events were predominantly mild to moderate and generally transient [60]. The rate of discontinuation due to adverse effects was similar between XT (27.6%) and the placebo (22.7%); however, this discontinuation rate warrants consideration in clinical practice, as it suggests that a meaningful proportion of patients may not tolerate the gastrointestinal side effects despite their transient nature [63].

The most profound clinical advantage of XT is its lack of D2-receptor-mediated side effects. Metabolic neutrality: Unlike typical and atypical antipsychotics, XT treatment was not associated with significant weight gain or extrapyramidal symptoms (EPS) [63]. Motor sparing: Rates of extrapyramidal symptoms, akathisia, and Parkinsonism were extremely low and comparable to the placebo [60,63]. No hyperprolactinemia: XT did not increase the prolactin levels, avoiding complications such as sexual dysfunction [38]. While generally safe, XT was associated with statistically significant increases in heart rate (9–10 beats per minute) [39,63]. Although this increase did not accompany significant QTc prolongation, it may still be clinically relevant in high-risk populations, particularly patients with pre-existing cardiovascular disease, arrhythmias, or autonomic dysfunction, as heart-rate elevation is a recognized concern with antipsychotic therapy [65]. These changes warrant monitoring in patients with pre-existing cardiovascular conditions.

It is important to note that the current evidence base is derived from controlled clinical trials with a maximum duration of 52 weeks in open-label extension. Long-term real-world data on cardiovascular safety, hepatic outcomes, and potential receptor desensitization remain to be established through post-marketing surveillance and observational studies. Furthermore, the generalizability of results from inpatient trial settings to outpatient clinical practice has not yet been confirmed.

## 9. The Competitive Landscape: Successes and Failures

The success of xanomeline–trospium (XT) has validated the M1/M4 muscarinic receptor agonist mechanism, but the path for follow-on compounds has been fraught with challenges. Emraclidine is a highly selective positive allosteric modulator of the M4 receptor [66]. Theoretically, allosteric modulators offer advantages over orthosteric agonists like xanomeline: they maintain the spatial and temporal fidelity of physiological signaling (activity-dependence) [66,67]. In a Phase 1b trial, emraclidine demonstrated clinically meaningful and statistically significant improvements in PANSS total scores at two dose levels (30 mg once daily, Cohen’s d = 0.68; 20 mg twice daily, Cohen’s d = 0.59) [66]. However, in November 2024, AbbVie announced that emraclidine failed to meet the primary endpoint in both Phase 2 EMPOWER-1 and EMPOWER-2 trials, showing no statistically significant separation from the placebo on the PANSS total score reduction at week 6. This Phase 2 failure was a significant setback for the selective M4 PAM approach and may suggest that the dual M1/M4 activity of xanomeline provides a synergistic benefit—M1 for cognition and M4 for dopamine modulation—that selective M4 PAMs lack, although other explanations including dosing, trial design, and pharmacokinetic differences cannot be excluded [67]. While Trace amine-associated receptor 1 (TAAR1) activation offers a selective non-dopaminergic approach, M1 and M4 muscarinic receptor activation may influence multiple nodes of the cortico-striato-thalamo-cortical circuit [68]. M1 signaling can enhance cortical excitability and support cognitive processing through effects on pyramidal neurons and interneuronal networks, whereas M4 activation may indirectly suppress mesolimbic dopaminergic activity via striatal cholinergic and dopaminergic pathways [68,69]. In this sense, muscarinic receptor activation may provide broader circuit-level modulation than selective non-dopaminergic agents such as TAAR1 agonists or glutamatergic enhancers.

Ulotaront (SEP-363856) acts via agonism of the Trace Amine-Associated Receptor 1 and serotonin 1A receptor, offering another non-D2 mechanism to modulate dopamine circuitry [70]. Like emraclidine, it demonstrated impressive Phase 2 results [71], but failed to separate from the placebo in the massive Phase 3 DIAMOND-1 and DIAMOND-2 trials [72]. The failure was attributed in part to an exceptionally high placebo response, potentially exacerbated by the COVID-19 pandemic environment during the trials [72], although other factors may have contributed. However, these repeated failures of novel mechanisms in late-stage trials underscore the unique robustness of the XT data, which showed consistent separation from the placebo across three independent Phase 3 studies. Iclepertin inhibits the Glycine Transporter 1, increasing synaptic glycine levels to potentiate N-methyl-D-aspartate receptor function [73]. Iclepertin is being developed as an adjunct specifically for cognitive impairment associated with schizophrenia. While Phase 2 data showed cognitive benefits [73], the Phase 3 CONNEX program (comprising three randomized controlled trials) did not meet the primary endpoint of significant improvement on the MATRICS Consensus Cognitive Battery composite score compared to the placebo, highlighting the immense difficulty of translating glutamatergic mechanisms into reliable clinical cognitive enhancement (Table 2) [36].

## 10. Precision Medicine: Biomarkers and Inflammation

The heterogeneity of schizophrenia has long plagued its treatment [74]. The identification of the Muscarinic Receptor Deficit Schizophrenia subgroup represents a promising step. Approximately 25% of patients exhibit a profound loss (>75%) of cortical M1 receptors [75].

These patients may represent a distinct biotype characterized by altered gene expression profiles in pathways interacting with the M1 receptor [76]. Developing neuroimaging ligands (PET tracers) or peripheral biomarkers to identify this subgroup and the level of their muscarinic deficit is a critical research priority to stratify patients for Cobenfy^®^ treatment [77]. Recent studies support the existence of a subgroup of individuals with schizophrenia who exhibit muscarinic receptor deficits [77,78]. Accordingly, muscarinic receptor-targeted therapies are being investigated alongside biomarker approaches to identify patients most likely to respond [77]. However, these biomarkers remain investigational and require further validation before they can be implemented into routine clinical practice. Emerging research links schizophrenia to systemic and neuroinflammation, involving microglial activation and elevated cytokines like interleukin-6 and tumor necrosis factor-α [79]. The vagus nerve regulates immune responses via the “cholinergic anti-inflammatory pathway”, involving macrophages via α7 nicotinic and M1 muscarinic receptors [80]. Xanomeline has been shown to activate this pathway, suppressing lethal inflammation in preclinical sepsis models [81]. This could be particularly relevant for the inflammatory subgroup of patients who often exhibit treatment resistance and severe cognitive deficits. However, it should be noted that peripheral muscarinic blockade by trospium could theoretically attenuate some of the peripheral anti-inflammatory actions of xanomeline, an interaction that has not been formally studied.

## 11. Pharmacoeconomics: Value and Access

The pharmacoeconomic profile of xanomeline–trospium (Cobenfy^®^) has been characterized by two independent analyses. The Institute for Clinical and Economic Review (ICER) calculated an incremental cost-effectiveness ratio of approximately $163,000 per quality-adjusted life-year (QALY) gained, falling within the upper range of conventional willingness-to-pay thresholds ($100,000–$150,000/QALY), with the favorable outcome contingent on the assumption that xanomeline–trospium carries no excess metabolic syndrome risk beyond the general population baseline [82]. This assumption has since been substantiated by long-term open-label data from EMERGENT-4 and EMERGENT-5, which demonstrated no clinically significant weight gain and no statistically significant rates of metabolic syndrome with up to 52 weeks of treatment [20,23], and no ICER access and affordability alert was issued for xanomeline–trospium.

Bjerke et al.subsequently conducted a 3-year budget impact analysis from the perspective of a hypothetical 1 million-member U.S. health plan, using the approved wholesale acquisition cost of $1850 per pack and modeling xanomeline–trospium as a second-line treatment at market uptake of 1%, 2%, and 3% in years 1 through 3, respectively [83]. The estimated per member per month (PMPM) budget impact was $0.0177 in year 1, rising to $0.1006 in year 3—a minimal incremental cost to payers. Critically, xanomeline–trospium’s motor-sparing and metabolically neutral profile generated a reduction in chronic adverse event management costs of approximately $59,700 over 3 years, driven by substantially lower rates of tardive dyskinesia (0.3% annually versus 0.5–4.8% for comparators) and metabolic syndrome (2.9%, equivalent to the general population baseline, versus 14.1–43.1% for traditional second-generation antipsychotics). These cost offsets are likely conservative, as the model excluded the costs of vesicular monoamine transporter 2 (VMAT2) inhibitors—the current first-line treatment for tardive dyskinesia—whose 30-day wholesale acquisition cost ranges from $5000 to $8000, representing a substantial further unquantified saving attributable to xanomeline–trospium’s motor-sparing profile.

Traditional second-generation antipsychotics such as olanzapine incur significant long-term costs attributable to metabolic syndrome, type 2 diabetes ($12,238 per patient annually), and cardiovascular disease ($20,382 per patient annually); by avoiding these sequelae, xanomeline–trospium generates meaningful cost offsets that become increasingly pronounced over longer time horizons and would be expected to further strengthen its economic case in lifetime modeling frameworks. A caveat applicable to both analyses is that drug costs were modeled at wholesale acquisition cost without rebates or Medicaid discounts; given that 47.9% of the modeled population carries Medicaid coverage—which mandates a minimum 23.1% discount from the average manufacturer price for branded drugs—the net budget impact to payers is likely lower than reported. Taken together, these data support the inclusion of xanomeline–trospium on health plan formularies as a second-line treatment for adults with schizophrenia, with a pharmacoeconomic profile that is competitively positioned relative to both branded and generic second-generation antipsychotics [83]. Notwithstanding these favorable modeled estimates, real-world cost-effectiveness remains uncertain, as long-term adherence, discontinuation rates, and downstream healthcare utilization have not yet been fully characterized in routine clinical practice.

## 12. Discussion

The evidence reviewed in the preceding sections demonstrates that the approval of xanomeline–trospium represents a clinically meaningful advance in schizophrenia pharmacotherapy. By validating a mechanism of action independent of D2 receptor blockade, xanomeline–trospium has expanded the therapeutic armamentarium. The consistent efficacy signals across the three EMERGENT Phase 3 trials, with effect sizes comparing favorably to existing antipsychotics, provide a robust evidence base [84]. The distinct safety and tolerability profile of xanomeline–trospium may provide patients with better long-term treatment adherence and more treatment options if they are unable to tolerate the metabolic and motor side effects of antipsychotics (Table 3).

However, several important limitations and evidence gaps must be acknowledged. First, all pivotal trials were placebo-controlled and conducted in inpatient settings. The absence of active-comparator trials precludes direct conclusions about the relative efficacy and tolerability of xanomeline–trospium compared to established antipsychotics. Due to the lack of head-to-head trials with standard antipsychotics, it is not possible to directly compare the efficacy and tolerability of xanomeline–trospium with those agents, making it difficult to determine its place within current treatment algorithms for schizophrenia. Real-world effectiveness in diverse outpatient populations remains to be established. Second, the cognitive benefits suggested by post hoc analyses did not reach statistical significance in the primary sample-wide analysis, and prospectively designed cognitive trials are needed. Third, the strict requirement to administer Cobenfy^®^ on an empty stomach, essential to preserve trospium’s peripheral muscarinic blockade, which food reduces by up to 85%, combined with gastrointestinal adverse events, contributed to approximately 28% treatment discontinuation in clinical trials, representing the primary real-world adherence challenge for this otherwise pharmacologically innovative combination. Fourth, the contraindications in moderate-to-severe hepatic and renal impairment may limit the eligible patient population, particularly given the high prevalence of metabolic comorbidities in schizophrenia. Fifth, clinically relevant drug–drug interactions via CYP3A4 and P-glycoprotein inhibition require careful management in a population frequently receiving multiple medications. Finally, long-term safety data beyond 52 weeks, including potential receptor desensitization and rare adverse events, remain to be characterized in real-world settings.

A limitation of the current evidence base is that the pivotal EMERGENT trials were conducted exclusively in inpatient settings. These settings provide structured medication administration, close monitoring, and frequent clinical assessment that may not reflect routine outpatient care. In real-world practice, patients must adhere to fasting requirements, manage gastrointestinal adverse effects, and navigate medical and psychiatric comorbidities that may affect treatment persistence and effectiveness. Prospective studies in outpatient and real-world settings are therefore needed to determine whether the benefits observed in clinical trials are maintained under routine clinical conditions.

## 13. Conclusions and Future Directions

The approval of xanomeline–trospium represents a significant development in the treatment of schizophrenia, as it establishes muscarinic receptor modulation as a viable therapeutic strategy for patients with schizophrenia, offering potential clinical benefits and expanding treatment options. The drug’s ability to improve positive and negative symptoms without the metabolic or motor burden of current therapies addresses important unmet needs in the field. The unique bitopic binding of xanomeline, coupled with the pharmacokinetic innovation of peripheral blockade, exemplifies a new era of rational drug design. However, the path forward requires vigilance. The failures of other novel agents like emraclidine and ulotaront highlight the fragility of psychiatric drug development and the confounding influence of placebo responses.

Beyond clinical efficacy, the molecular pharmacology of xanomeline provides a mechanistic basis for its distinctive profile. Cryo-EM structural studies have revealed that xanomeline engages M4 muscarinic receptors through a bitopic binding mode, simultaneously occupying the orthosteric acetylcholine pocket and an adjacent extracellular allosteric vestibule. This dual anchorage slows dissociation (reduced k_off), producing wash-resistant receptor engagement that persists beyond the compound’s ~5-h plasma half-life and contributes to sustained efficacy. The preferential activity at M1 and M4 subtypes (8–50-fold over M2/M3/M5) further enables central target engagement while limiting peripheral cholinergic activation. These molecular insights validate muscarinic agonism not as a broad cholinergic stimulus, but as a subtype-biased, bitopic pharmacological strategy; a template for future GPCR-targeted CNS drug design.

To capitalize on this momentum, future research must prioritize long-term vigilance regarding receptor desensitization and rare adverse events in multi-year real-world settings, alongside the validation of biomarkers to operationalize the Muscarinic Receptor Deficit Schizophrenia concept for precision treatment. Concurrent efforts should explore combination strategies, evaluating xanomeline–trospium as an augmentation for clozapine-refractory patients or in concert with glutamatergic modulators. Head-to-head trials comparing xanomeline–trospium with standard antipsychotics are urgently needed to establish its position in treatment algorithms. Additionally, prospectively designed trials with validated cognitive endpoints should be prioritized to evaluate the M1-mediated cognitive benefits suggested by preliminary analyses. Ultimately, M1/M4 muscarinic receptor agonist therapy represents not merely a novel drug class, but a significant advance toward a circuit-based, precision medicine approach capable of treating the underlying disorder of schizophrenia rather than solely its psychotic manifestations.

## Figures and Tables

**Table 2:** Comparative pharmacological profiles: First-generation antipsychotics, second-generation antipsychotics, and xanomeline–trospium.

Xanomeline–Trospium (Cobenfy^®^)	Second-Generation Antipsychotics (SGAs)	First-Generation Antipsychotics (FGAs)	Feature
Xanomeline (agonist) + Trospium chloride (peripheral antagonist)	Olanzapine, Risperidone, Quetiapine, Clozapine, Aripiprazole	Haloperidol, Chlorpromazine, Fluphenazine	Representative agents
Dual M1/M4 muscarinic receptor agonism (bitopic binding). No direct D2 blockade.	D2 receptor antagonism/partial agonism + multi-receptor activity (5-HT_2A_, H_1_, α_1_)	High-affinity D2 receptor antagonism	Primary mechanism of action
Effective. PANSS Positive LSM Δ −3.2 (*p* < 0.0001). Cohen’s d 0.60–0.65 across EMERGENT trials [62].	Effective. Comparable or modestly superior to FGAs in some analyses.	Effective. Strong D2 occupancy reduces psychosis.	Efficacy: Positive symptoms
Significant improvement. PANSS Negative LSM Δ −1.7 (*p* < 0.0001). M1-mediated cortical restoration [62].	Modest improvement (5-HT_2A_ mediated). Clozapine shows greatest benefit. Overall limited efficacy.	Limited. D2 blockade may worsen secondary negative symptoms (neuroleptic dysphoria).	Efficacy: Negative symptoms
Preliminary signal: Numerical improvement (*p* = 0.16 sample-wide); significant in cognitively impaired subgroup. Dedicated trials needed [33].	Minimal to no clinically meaningful benefit despite theoretical 5-HT mechanisms.	No benefit. D2 blockade does not address prefrontal hypofunction.	Efficacy: Cognitive symptoms
Comparable to placebo. No D2 occupancy = no EPS, no akathisia, no tardive dyskinesia risk [62,65].	Lower risk than FGAs but still present, especially risperidone at higher doses. Aripiprazole: Akathisia. Tardive dyskinesia remains a concern [27].	High risk. Dose-dependent EPS, akathisia, dystonia, Parkinsonism. Tardive dyskinesia with chronic use [25,26,27].	Motor side effects (EPS)
Metabolically neutral. No significant weight gain. No dyslipidemia or insulin resistance observed in EMERGENT trials up to 52 weeks [65].	Major concern. Olanzapine/clozapine: significant weight gain, dyslipidemia, insulin resistance, new-onset diabetes. Metabolic syndrome rates 30–50% [8,23].	Low-potency FGAs (chlorpromazine): Moderate weight gain. High potency (haloperidol): Minimal metabolic effects.	Metabolic profile
No elevation. No D2 tuberoinfundibular blockade. Prolactin levels comparable to placebo [33].	Variable. Risperidone/paliperidone: High. Aripiprazole: May lower prolactin. Olanzapine/quetiapine: Transient/mild.	Significant. Sustained hyperprolactinemia causing gynecomastia, galactorrhea, amenorrhea, sexual dysfunction, osteoporosis.	Prolactin elevation
Minimal. No H_1_ or α_1_ blockade. Somnolence rates comparable to placebo [65].	Olanzapine, quetiapine, clozapine: marked sedation. Aripiprazole: less sedating.	Low-potency FGAs: significant (H_1_ blockade). High potency: less sedating.	Sedation
Gastrointestinal: Nausea (19%), constipation (21%), dyspepsia (19%), vomiting (14%). Predominantly mild/moderate and transient. Heart rate increase (9–10 bpm) [43,65].	Weight gain, dyslipidemia, sedation, diabetes risk, QTc prolongation (some agents), agranulocytosis (clozapine).	EPS, akathisia, tardive dyskinesia, hyperprolactinemia, QTc prolongation (some agents), sedation.	Primary adverse events
No QTc prolongation. Heart rate increase (9–10 bpm); monitoring recommended. No orthostatic hypotension [35,60].	QTc prolongation (ziprasidone). Orthostatic hypotension (clozapine, quetiapine). Myocarditis (clozapine).	QTc prolongation (haloperidol IV, thioridazine, pimozide). Orthostatic hypotension (low-potency agents).	Cardiovascular risk
Compartmentalized design: Xanomeline acts centrally (crosses BBB). Trospium (quaternary ammonium) blocks peripheral muscarinic receptors to mitigate systemic toxicity [41,42,43].	Central multi-receptor action. Peripheral effects (metabolic, autonomic) are integral to mechanism.	Central D2 blockade only. Peripheral effects are unmitigated off-target consequences.	Pharmacokinetic strategy
Must dose 1 h before/2 h after meals. Contraindicated in moderate–severe hepatic/renal impairment. GI tolerability. No active-comparator trials yet. CYP3A4/P-gp interactions [35,59].	Metabolic syndrome. Clozapine: Agranulocytosis monitoring (REMS). Weight gain reduces adherence.	Narrow therapeutic window. EPS limits tolerability and adherence. Tardive dyskinesia risk with long-term use.	Key limitations

**Table 3:** The landscape of novel non-dopaminergic therapeutics.

Important Notes	Clinical Status & Outcome	Mechanism of Action	Therapeutic Agent
Dual M1/M4 action may give synergistic efficacy that selective agents lack.	Approved. Showed success in EMERGENT-1, 2, and 3 trials.	Dual M1/M4 Agonist. Utilizes “bitopic” binding and peripheral blockade.	Xanomeline–trospium (Cobenfy^®^)
Maintains spatial and temporal fidelity of physiological signaling. Phase 2 failure may reflect dosing, trial design, or limitations of selective M4 approach.	Phase 1b showed efficacy (Cohen’s d = 0.59–0.68). Phase 2 EMPOWER-1 and EMPOWER-2 failed to separate from placebo (November 2024).	M4-Selective PAM. Acts like a brake on dopamine.	Emraclidine
Failure attributed in part to high placebo response; other factors may have contributed.	Failed to separate from placebo in DIAMOND-1 and DIAMOND-2.	TAAR1/5-HT1A agonist. Modulates dopamine firing rates without blocking D2.	Ulotaront (SEP-363856)
Challenge: translation of glutamatergic mechanisms into reliable clinical cognitive benefits.	Phase 2 showed cognitive benefits. Phase 3 CONNEX program (three trials) did not meet the primary endpoint.	GlyT1 Inhibitor. Increases synaptic glycine to potentiate NMDA function.	Iclepertin

## Data Availability

Data sharing is not applicable to this article as no new data were created or analyzed in this study.

## List of Non-Standard Abbreviations

ACh: Acetylcholine; AUC: Area Under the Curve; cAMP: Cyclic Adenosine Monophosphate; Cmax: Maximum Plasma Concentration; D2: Dopamine 2 Receptor; eGFR: Estimated Glomerular Filtration Rate; EPS: Extrapyramidal Symptoms; GlyT1: Glycine Transporter 1; GPCRs: G-Protein-Coupled Receptors; KarXT: Investigational name for xanomeline–trospium (Karuna Therapeutics/BMS); approved as Cobenfy^®^, LSM: Least Squares Mean; MRDS: Muscarinic Receptor Deficit Schizophrenia; MSNs: Medium Spiny Neurons; PANSS: Positive and Negative Syndrome Scale; PANSS-EC: Positive and Negative Syndrome Scale-Excited Component; PV: Parvalbumin; TAAR1: Trace Amine-Associated Receptor 1; XT: Xanomeline–Trospium.
